# Supporting Children with Disabilities in Low- and Middle- Income Countries: Promoting Inclusive Practice within Community-Based Childcare Centres in Malawi through a Bioecological Systems Perspective

**DOI:** 10.1007/s13158-018-0223-y

**Published:** 2018-07-04

**Authors:** Mike McLinden, Paul Lynch, Anita Soni, Alfredo Artiles, Foster Kholowa, Elizabeth Kamchedzera, Jenipher Mbukwa, Mika Mankhwazi

**Affiliations:** 1 School of Education, University of Birmingham, Edgbaston, Birmingham, B15 2TT UK; 2 Graduate College, Arizona State University, PO Box 37100, 3151, Phoenix, AZ 85069 USA; 3 University of Malawi (Chancellor College), PO Box 280, Zomba, Malawi

**Keywords:** Early childhood development, Inclusion, Disabilities, Bioecological systems, Malawi

## Abstract

Given the narrow scope and conceptualisation of inclusion for young children with disabilities in research within low- and middle-income countries (LMICs) contexts, we draw on a bioecological systems perspective to propose the parameters for a broader unit of analysis. This perspective situates human development within a specific cultural context in which family, peers and schooling are regarded as key in responding to young children with disabilities in a given setting. We outline a new bioecological model to illustrate the proximal and distal factors that can influence inclusive early development for children with disabilities within LMICs. To illustrate the relevance of this model to early child development research, we consider its application, as a conceptual framework, with reference to a research study in Malawi. The study was designed to promote greater inclusive practice for young children with disabilities in Community-Based Childcare Centres (CBCCs) with a particular focus on the role of the CBCC volunteer ‘caregiver’ in rural Malawi. It has significance for educators, service providers and researchers concerned with facilitating inclusive early development across national boundaries and contexts.

## Résumé

L’étroitesse de la portée et de la conceptualisation de l’inclusion de jeunes enfants handicapés dans le corpus de recherche dans les pays à revenu faible et (PRFI) contextes intermédiaire, nous puisons dans une perspective de systèmes bioécologiques pour proposer des paramètres pour une unité d’analyse plus large. Notre perspective situe le développement humain dans un contexte culturel spécifique dans lequel la famille, les pairs et la scolarité sont considérés comme des éléments clés pour apporter une réponse à la question des jeunes enfants handicapés dans un contexte donné. Nous décrivons un nouveau modèle bioécologique pour illustrer les facteurs proximaux et distants qui peuvent influencer le développement des enfants handicapés dans les PRFI pendant leurs premières années. Pour illustrer la pertinence de ce modèle pour la recherche sur le développement des jeunes enfants, nous prenons en considération son application comme cadre conceptuel, en référence à une étude menée au Malawi. L’étude a été conçue aboutir de promouvoir des pratiques plus inclusives pour les enfants handicapés fréquentant des garderies communautaires, en mettant un accent particulier sur le rôle de l’«assistant » bénévole dans les zones rurales du Malawi. Elle est importante pour les éducateurs, les prestataires de services et les chercheurs qui veulent faciliter un développement inclusif des jeunes enfants, au-delà des frontières et contextes nationaux.

## Resumen

Debido al poco alcance y la conceptualización limitada de la inclusión de niños de edad temprana con discapacidades en investigaciones realizadas en el contexto de países de ingresos bajos y medios, utilizamos una perspectiva de sistemas bio-ecológicos, con el fin de proponer parámetros más amplios de análisis. Esta perspectiva sitúa al desarrollo humano dentro de su contexto cultural específico en el cual la familia, los compañeros y la escuela son considerados como factores vitales en la atención a niños de edad temprana con discapacidades. Se describe un modelo bio-ecológico para ilustrar los factores proximales y distales que pueden influenciar el desarrollo temprano en contextos inclusivos para niños con discapacidades en países de ingresos bajos y medios. Con el fin de ilustrar la importancia de este modelo para la investigación del desarrollo infantil temprano, consideramos su aplicación, como marco conceptual, con referencia a una investigación llevada a cabo en Malaui. El estudio se diseñó con el objeto de promover mejores prácticas inclusivas para niños pequeños con discapacidades en Centros Infantiles Comunitarios (CICs) con un enfoque especial en el papel que juega el cuidador voluntario del CIC en zonas rurales de Malaui. Este estudio es relevante para educadores, centros de educación e investigadores interesados en brindar desarrollo temprano inclusivo en diferentes contextos y territorios.

## Introduction

Early childhood development is considered to be a significant phase of growth and development which influences outcomes across an individual’s entire life and provides an important period of opportunity and a foundation for lifelong learning and participation (World Health Organisation 2012). Over the past 15 years, global interest in promoting early childhood development has increased significantly with emerging evidence for the effectiveness of combined sector programmes (e.g. education, health, stimulation, protection and nutrition), particularly if provided in the first 1000 days of life (Black et al. 2017). The UN Convention on the Rights of the Child (United Nations 1989) and the Convention on the Rights of Persons with Disabilities (United Nations 2006) affirm that all children have the right to develop to their full potential and that governments should guarantee that young children with disabilities receive high-quality education.

The increased international emphasis on ensuring access to quality early childhood development services for young children can be demonstrated through the UN Sustainable Development Goals (SDGs) which map out in an ambitious agenda for sustainable development over the next 12 years (United Nations 2015). SDG 4 seeks to ‘ensure inclusive and equitable quality education and promote lifelong learning opportunities for all’ (United Nations 2015) and includes an outcome target (4.2) to ‘ensure that all girls and boys have access to quality early childhood development, care and pre-primary education so that they are ready for primary education’ (p. 21). The wording of this target ensures that equality of opportunity and access to *quality* provision is considered for all young children, including those with disabilities. Access to quality early childhood development services for these young children is considered to be particularly important given a need for structured opportunities that include stimulation and development of key functional skills (e.g. WHO 2012).

Different international contexts (including those in low-income settings) are expected to promote quality early childhood development, but may not have sufficient resources to ensure, for example, adequate inspection and monitoring of programmes. In this article, we propose the parameters of a bioecological model to examine the multi-layered influences on replicating and scaling up quality early childhood development in low- and middle-income countries (LMICs) and consider its application to research design in the context of Malawi.

We begin the article with an analysis of the scope of early childhood development research within LMICs that has had a focus on the inclusion of young children with disabilities. We highlight the dearth of research in particular disability areas as well as the limited consideration of the interactions with broader social and cultural influences on development. We then introduce Bronfenbrenner’s bioecological systems theory of human development (e.g. Bronfenbrenner 2005) and examine how this theory offers a helpful conceptual reference point for examining inclusive education in the literature. We draw upon recent applications of Bronfenbrenner’s work with respect to early childhood development and inclusive education, to propose the parameters of a new model of inclusive early childhood development with a particular focus on children with disabilities in LMICs. To illustrate the relevance of the bioecological model to early childhood development research, we consider its application as a conceptual framework for a research study (*Let’s Grow Together*) that is seeking to provide the Malawi Government and its partners in education with a better understanding of the complex dynamics that can enable or inhibit quality early childhood development for young children with disabilities.

The study is designed to promote greater inclusive practice for young children with disabilities in Community-Based Childcare Centres (CBCCs) with a focus on the role of the CBCC ‘caregiver’ (a volunteer adult appointed to run the centre). Through drawing on a bioecological conceptual lens, we emphasise the significance of ensuring the research focus is on the ‘interrelatedness’ between the development of ‘active’ young children with disabilities and their respective learning environments. We conclude the article by highlighting the potential relevance of a bioecological systems perspective for future inclusive early childhood development research and policy development, arguing that situating research studies within the parameters of such a framework enables potential comparison of studies across national boundaries and contexts.

### Conceptualisation of Inclusive Early Childhood Development in LMICs

As reported by World Health Organisation (WHO 2012), inclusive early years experiences prior to starting school offer children with disabilities critical space to ensure optimal development by providing them with opportunities for child-focused learning, play, communication activities and peer interaction. Inclusive early childhood development should therefore ensure that children with disabilities receive specialised health care and that families of children with disabilities are able to access basic and essential social services in their communities (UN Children’s Fund 2012). This will be particularly true in LMICs, which are defined by the World Bank (2018) as countries which have gross national income per capita ranging from: *Low* < $1005, *Lower middle* from $1006 to $3955 and *Upper middle* from $3956 to $12,235. Recent research highlights the wide range of factors that can serve as potential barriers for young children with disabilities in LMICs in achieving access to services (e.g. Lynch et al 2018; Gladstone et al. 2017), and many of these children, particularly those with non-severe disabilities, may not be identified until they reach school age (Cunningham 2004).

Systems for early identification are few and are often underdeveloped in rural parts of many countries in sub-Saharan Africa, resulting in missed opportunities to identify those children at significant risk of developmental delay and to prevent issues, such as a loss of confidence in parenting skills (Cunningham 2004). Only a few studies have assessed programmes in LMICs that specifically target early childhood development for children with neuro-developmental delays or disabilities, with limited research evidence available for programmes that have as their focus specific disabilities (e.g. sensory impairments, motor impairments, behavioural and communication difficulties and learning difficulties), all of which may have different aetiologies and may require specific interventions (Yousafzai et al. 2014). In the light of the fact that the majority of a young child’s life may be spent at home and in early childhood development settings, rigorous and methodologically sound studies are required to analyse the capacity and role of parents as well as those who have the responsibility for caring and educating children with disabilities in early childhood development settings.

There is a wealth of literature on early childhood development that emphasises the importance of acknowledging a given cultural context in which family, peers and schooling are regarded as key in responding to children with early neuro-developmental delays and disabilities. These contexts have evolved over time at multiple levels and in particular historical and political contexts (Albrecht et al. 2001). Understanding the nature of contextual influences on early development is considered to be influential in research design and in particular when formulating the focus for analysis within a given study, to ensure it is not just on the child in isolation, but rather seeks to capture the nature of the broader context within which development takes place. As an example, Skinner and Weisner (2007) highlight the importance of the sociocultural context of development when researching children with disabilities, noting that when sociocultural theorists conceptualise a young child, ‘they do not think of a child as an autonomous individual floating in space. Rather, they think of that child somewhere, surrounded by social context, ecology, resources, local meanings and understandings, and the possible life pathways available’ (p. 302).

Such a perspective is supported by Artiles and Kozleski (2016) who conclude an analysis of literature on inclusive education by arguing that future research should include broadening the unit of analysis to ‘systems of activities’ (p. 2), as well as documenting processes and outcomes. They report that most studies in the literature analysis had either a whole school or a classroom focus with the *individual* student in mind. As such, they argue that research should be grounded in a unit of analysis that examines individuals ‘embedded in multi-layered systems of activities’ that take into account the institutional conditions under which students participate in inclusive systems, thereby enabling ‘scholars to link systematically macro and micro forces in the study of inclusion’ (Artiles and Kozleski 2016, p. 18).

Werning et al. (2016) contend that finding ways to commit to locally situated inclusive education contexts is critical for its successful implementation. In reflecting on the ‘future’ of inclusive education, they outline three recommendations for inclusive education research and practice in both high- and low-income countries:

- Use of situated models;
- Consideration of the importance of educational quality in the process of realising inclusive education;
- Creating positive pressure.

We examine next how Bronfenbrenner’s bioecological systems theory of human development provides the basis for developing an ecological model of inclusive education in early years settings. We outline the parameters of a new ‘situated model’ of inclusive early childhood development within LMICs that reflects these recommendations through acknowledging broader sociocultural factors and through which the importance of ‘educational quality’ within early childhood development settings can be promoted to create ‘positive pressure’ in the context of a given country.

### An Ecology of Inclusive Early Childhood Development

The bioecological systems theory of human development was proposed by Uri Bronfenbrenner to understand the multi-layered influences on human development within the complex ‘ecology’ within which individuals live. It was originally framed as an ‘ecological’ systems theory (e.g. Bronfenbrenner 1977), but later adapted to reflect the importance of the individual at the centre of the complex ecology through reference to the term ‘*bio*ecological’ (e.g. Bronfenbrenner 2005). As noted by Anderson et al. (2014), this distinction in terminology is of relevance to a consideration of the construct of inclusive education, ‘as it is precisely the characteristics of the learner that should not influence whether or not a student is delivered an effective IE. It is, however, the environments and factors that sit within these, along with the relationships and interconnections between them that influence the success (or not) of IE’ (pp. 5–6).

The ‘cornerstone’ of the ecological systems theory was defined by Bronfenbrenner as being: ‘the scientific study of the progressive, mutual accommodation, *throughout the life course*, between an active, growing human and the changing properties of the immediate settings in which the developing person lives, as this process is affected by the relations between these settings, and by the larger contexts in which the settings are embedded’ (Bronfenbrenner 2005, p. 107, original italics). Within the context of human development, the theory is commonly represented as a nested system of ‘environments’ often illustrated as a series of concentric circles situated around a developing individual (e.g. Coleman 2013; Anderson et al 2014; Rogoff 2003; McLinden et al. 2016; Hewett et al. 2017). Each circle refers to nested but separate systems to reflect the complex ecology in which an individual develops.

The individual at the centre of the ecology is viewed as being an ‘active’ agent in development, and as reported by Hewett et al. (2017), the ‘context’ in which this takes place is described by Bronfenbrenner with reference to the five interrelated systems:

- *microsystem*—factors in the environment immediately around the individual;
- *mesosystem*—interactions between factors within the microsystems;
- *exosystem*—factors outside the individual’s immediate environment that impact upon their development;
- *macrosystem*—factors and culture outside the physical environment;
- *chronosystem*—human development over time.

McLinden et al. (2016) report that Bronfenbrenner’s bioecological systems theory has been drawn upon extensively in the literature for analysing the multi-layered influences (i.e. proximal and distal) on child development (e.g. Ertem 2011; Rogoff 2003; Coleman 2013). As an example, Coleman (2013) argues that the theory provides a ‘lens’ through which to appreciate ‘multiple sources of influence and interconnection’ (p. 47), with Rogoff (2003) reporting that a key strength of the theory is that it ‘emphasises studying the relations among the multiple settings in which children and their families are directly and indirectly involved’ (p. 48).

There is also increasing evidence in the literature to indicate the value of adopting such a framework in order to analyse inclusive practice in the contexts of school education (e.g. Anderson et al. 2014; McLinden and McCracken 2016) as well as in higher education (e.g. Hewett et al. 2017; McLinden et al. 2018). As an example, in considering the relevance of Bronfenbrenner’s systems theory to ‘inclusivity’ in higher education, Hewett et al. (2017) outline a ‘Bioecological Model of Inclusive Education’ to examine the experiences of students with vision impairment in the UK. They report that applying Bronfenbrenner’s theory to develop such a model provides ‘a valuable framework, allowing the researcher to take a more holistic view of the learner’s experience in their immediate and broader context, and the progressive mutual accommodation between learner and educator’ (p. 108). Similarly, in proposing an ecological model of ‘inclusive education’ in schools, Anderson et al. (2014) report that Bronfenbrenner’s theory ‘offers an invaluable framework within which to organise the environmental factors and understand their influence on inclusivity by placing the learner at the centre’ with each contributory factor ‘located in relation to the learner’s educational ecosystem’ (p. 28). Three principles of IE for a learner in school education are outlined within this model (*participation*, *achievement* and *value*), with the authors arguing that *inclusive* education should ensure all children are able to:

- *participate* through being actively engaged in all aspects of schooling,
- *achieve* through access to appropriate learning goals that meet individual needs supported with meaningful and attainable assessment;
- be *valued* for who they are as an individual and what they have to offer, to others.

Whilst of value in highlighting the relationships between people and systems within a complex ecology of inclusive education, the model outlined by Anderson et al. (2014) has an explicit focus on primary and secondary schools and therefore whilst the factors they include in their ‘ecology’ have resonance when applied to early childhood development settings, there will also be important differences (e.g. curricula drawn upon to guide early childhood development practice, the professional background of the staff engaged in supporting the children, etc.). Further, there is no explicit consideration of contextual factors that might influence inclusive early childhood development in LMICs (e.g. potentially limited access to qualified staff, healthcare and social service facilities; limited recognition of the needs of children with disabilities).

An example of a model that has adopted such an approach is a topic guide on holistic, multi-sectoral early childhood development in low-resource settings (Woodhead et al. 2014) which explicitly draws on a bioecological model as a conceptual framework. Woodhead et al. (2014) argue that a bioecological perspective is helpful as a starting point for examining early childhood development in such settings given it offers a ‘systemic model that identifies multiple potential entry points and delivery platforms for early years development. The most obvious proximal entry points are the programmes in which young children participate. But the model also recognises distal entry points, including laws and regulations, social protection programmes, especially those that alter parents’ capacities to support their children’s development’ (p. 13). Further, they note that adding a timeline dimension in the form of the *chronosystem* ‘reinforces that early childhood development processes and systems are dynamic, and while some interventions are age-critical, others are more continuous’ (p. 13).

We draw on the ecological perspectives outlined above as conceptual reference points to propose the parameters of a new bioecological model that has as its primary focus inclusive early childhood development for young children with disabilities in LMICs. To illustrate the application of the bioecological model to early childhood development research in LMICs, we then examine its application as a conceptual framework through reference to a research study in Malawi.

### A Bioecological Model of Inclusive Early Childhood Development in LMICs

**Fig. 1:**
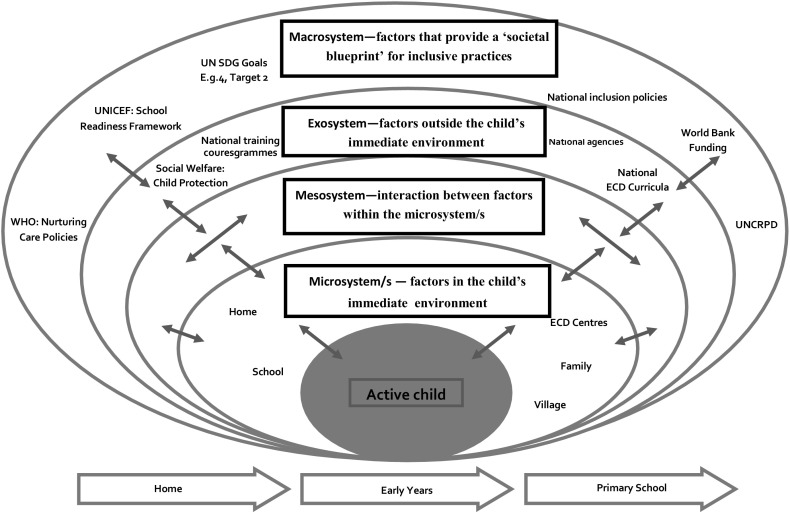
A bioecological model of inclusive early childhood development to illustrate the proximal and distal environmental factors that influence the inclusive education of young children with disabilities in LMICs. (based on Bronfenbrenner 2005; Anderson et al. 2014; Hewett et al. 2017; Woodhead et al. 2014)

A bioecological model of inclusive early childhood development is presented in Fig. 1 to illustrate the proximal and distal environmental factors that can influence the inclusive education of young children with disabilities in LMICs.

At the centre of the model is the ‘active’ young child. Each child will have distinctive characteristics and needs, and it will be important to acknowledge the nature and extent of these in any analysis of his or her participation in a given early childhood development programme or research study. Examples of proximal and distal environmental factors that can influence inclusive early childhood development are presented within each of the interrelated systems surrounding the child.

The* microsystem* incorporates those factors in the environment immediately around the young child. Examples include the settings in which the child directly experiences formal and informal learning (e.g. home, nursery/early childhood development setting), early childhood development setting and the staff and volunteers who work there, peers, the learning spaces, environment cultures and routines, resources and the play environments. It also includes community-based organisations which set up and manage early years centres, management committees and interactions with early childhood development coordinators.

The* mesosystem* incorporates interactions between factors within the *microsystem*. Examples of factors within this system include activities that take place to facilitate inclusion within early childhood development settings. These include the structures to support care and learning (e.g. coordination between different agencies, home-centre links) as well as the training of nursery school staff or volunteer child-carers who support the child’s care and learning.

The* exosystem* incorporates factors outside the child’s immediate environment that impact upon their development. Factors within the *exosystem* encompass the relationships and processes that take place between environmental settings. Of significance is that whilst these settings do not ordinarily contain the developing person, events occur that influence processes within the immediate setting that does contain that person (e.g. Bronfenbrenner 2005).

The* macrosystem* incorporates factors that provide ‘a societal blueprint for a particular culture, subculture, or other broader social context’ (Bronfenbrenner 2005, pp. 149–150). These include those environmental factors that influence inclusive early childhood development in a particular LMIC. It incorporates broader ‘global’, ‘political’, ‘social’ and ‘historical’ factors that together help to shape the blueprint for inclusive educational practice within a given context (e.g. Anderson et al. 2014). Examples of such factors in the current global context of early childhood development include the implementation of Article 24 of the UN Convention for the Rights of Persons with Disabilities (CRPD); UNICEF statements advocating ‘integrated early childhood development (UNICEF, 2012) and internationally agreed SDGs (UN 2015). Other factors include early childhood development national legislation and policy (e.g. how is it conceptualised and implemented at national, state and regional levels); national curricula (e.g. what national curricula are drawn upon to support early childhood development); early years curriculum structures, inclusive curriculum policies as well as funding models for inclusive policies.

The* chronosystem* acknowledges human development over time. The *chronosystem* equates with the different phases of early childhood development in the context of a given country context. Facilitating effective transition between different educational phases/settings is of particular relevance for children with disabilities and resonates with Bronfenbrenner’s notion of ‘ecological transitions’ (Bronfenbrenner 2005) as children move from one setting to another (e.g. home to early childhood development settings and then potentially to primary school).

### Application of the Inclusive Early Childhood Development Model to a Research Study in Malawi

Malawi is a relatively small country situated in South-East Africa. In line with the Convention of the Rights of Children, which was ratified and signed in 1991, Malawi, is implementing a comprehensive early childhood development programme which aims to enhance holistic development, especially in the areas of early learning, stimulation, health, education, protection, nutrition, hygiene and sanitation. There has been a rapid expansion of early childhood development provision, rising from 3% (2003) to 45% (2016) for approximately 3.7 million children (Malawi National Statistical Office 2016) with continued commitment to expand CBCCs over the next 10 years. Malawi was one of the first African countries to have a network of CBCCs for young children (3–5 years) supported by Ministry of Gender, Disability and Social Welfare (MGCDSW). Whilst CBCCs provide an early learning environment to children living nearby, it is reported that the *quality* of most of the CBCCs, measured in terms of buildings, sanitation facilities, staff numbers, capacity, materials and equipment, has fallen short of the early childhood development Monitoring and Evaluation Framework set out by the MGCDSW (Munthali et al. 2008).

The National Policy on Early Childhood Development (Malawi Government 2017) highlights that the multiple challenges faced by young children in Malawi can be attributed to the fact that provision of early childhood development services has often been fragmented and sets out a commitment to increasing the quality of early childhood development provision through the National Policy on Early Childhood Development. Access to early childhood development services is reported as being just over 45% with significant gaps in terms of access given approximately 55% of all eligible children do not access CBCCs. This policy notes that the situation is worse for children with ‘special needs’, children on the street and other vulnerable children’ (p. 22). The main challenges associated with service provision in early childhood development settings are an overreliance on volunteer caregivers who have low education attainment and have received little or no training in early childhood development. The World Bank (2015) through an Impact Evaluation Study reported one-third of caregivers in 199 CBCCs did not have a Primary School Leaving Certificates and less than 40% had received any training on early childhood development. Most CBCCs were not considered to be ‘child and disability friendly’, because ‘they do not have adequate material resources, regulated child development practitioners’ or ‘strong and effective monitoring and supervisory systems’ (World Bank 2015, p. 22).

Whilst there has not been extensive evaluation of the role of CBCCs, to date there is evidence that highlights the many challenges the community-based management committees face in providing quality early childhood development provision for young children in a given region, as well as highlighting the significance of ensuring there is appropriate training for the caregivers (e.g. Munthali et al. 2008; Neuman et al. 2014; Munthali et al. 2014).

Evidence from more recent studies in Malawi has demonstrated how, despite the strong interest to improve the quality of early childhood development programmes, one of the main challenges encountered is providing adequate support for parents and their children with disabilities (International Centre for Evidence in Disability 2014). As an example, Munthali et al. (2014) found that CBCCs were reluctant to enrol children with ‘special needs’ because of a ‘lack of appropriate training and resources’ (p. 4). Further, they report that caregivers did not register children who were unable to communicate ‘mainly because [they] may fail to interact well with his or her friends and caregivers’ (p. 5) and tended to turn away children who they considered to have behaviour problems. Furthermore, ensuring quality training to volunteer caregivers has been a major challenge for the Malawi Government and other service providers, particularly in the area of disability. An initial two-week training programme, following the *National Syllabus for Integrated Early Childhood Development* (Malawi Government 2008), is normally currently offered to caregivers across the country. Whilst this training programme contains brief input about the rights and legislation with respect to children with disabilities, it does not provide practical solutions to support these children when attending the CBCC or develop reflection on practice on how to do this.

To illustrate the application of the bioecological model outlined in Fig. 1 to early childhood development research in LMICs, we examine its application as a conceptual framework for a research study that seeks to provide the Malawi Government and its partners with a better understanding of the factors that can enable or inhibit quality early childhood development for children with disabilities in CBCCs.

### Research Study: ‘Let’s Grow Together’

‘Let’s Grow Together’ is a 3-year (2015–2018) multi-agency study that seeks to promote the inclusion of children with disabilities in CBCCs in a rural district of Southern Malawi. The main purpose of the project is to explore ways of developing the skills of caregivers to support children with disabilities in CBCCs through the use of inclusive strategies and resources. To achieve this, the study is training caregivers using an Inclusion Resource Pack, which is integrated into a *National Integrated Early Childhood Development Training Manual* used by nationally recognised agencies including the Association of Early Childhood Development Training Centre in Malawi (AECDM). A key output of the study is the development of a revised evidence-based curriculum for caregivers and the provision of inclusive learning materials for the Malawi Government to use as part of its national early childhood development training programme. The study also seeks to share evidence that will aid the Malawi Government (specifically the Ministry of Gender, Children, Disability and Social Welfare in collaboration with the Ministry of Education, Science and Technology) and key stakeholders (e.g. UNICEF, Open Society Foundation) to better understand the complex dynamics that ‘enable’ or ‘inhibit’ quality early childhood development for children with disabilities using a mixed-method research design in one rural district in Southern Malawi.

The main environmental settings of the study within the *microsystem* are the CBCCs in the selected region of Malawi. A *Community-Based Child Centre Rating Scale* has been developed that will be drawn upon to rate the quality of provision of the centres, as well the level of ‘participation of children with disabilities. This focus includes rating aspects of caregiver supervision, engagement with the children, routine and structure, managing children’s behaviour, social development and provision of children with disabilities.

**Fig. 2:**
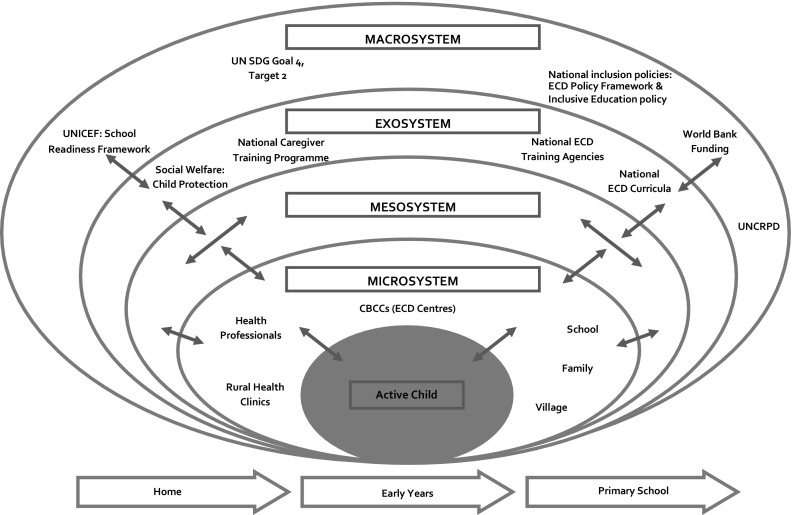
A bioecological model of early childhood development to illustrate the environmental factors that influence the inclusion of young children with disabilities within CBCCs in Malawi

The training of the caregivers is located in the *mesosystem*. This training is addressed through the development of a pilot ‘Inclusion Resource Pack’ (IRP) which includes basic information on understanding disability, early literacy and storytelling, early maths activities and which provides practical guidance on how to include young children with disabilities in the daily activities of a CBCC. Whilst the outer layers of the framework are considered to be outside of the child’s direct agency, they nevertheless have relevance to a broader context in which the CBCCs operate. As examples, the *exosystem* includes the inclusive practices of a given CBCC, as well as budget allocations to ensure learners with particular types of needs are suitably accommodated for at the setting. The *macrosystem* includes national early childhood development and education policies which contain guidelines on ways to include children with disabilities into schools. An illustration of the environmental factors that influence the inclusion of young children with disabilities in CBCCs is presented in Fig. 2.

## Discussion

A distinctive aspect of drawing on a bioecological systems theory for early childhood development research is its focus on the development of an ‘active’ young child whilst acknowledging the complexity and multi-dimensional nature of the changing influences on a child’s development over a given timeframe. As Tudge et al. (2009) report, it therefore emphasises the *interrelatedness* between the developing person and the context in which development takes place. Conceptualising the developing child at the centre of the framework therefore serves to emphasise the importance of recognising *individual* strengths and needs and of ensuring that as far as possible, the young child, regardless of the nature of his or her disabilities, has opportunities to be an ‘active’ participant in his or her learning.

With respect to such participation, the notion of *progressive* and *mutual* accommodation is of particular relevance as it suggests a need to ‘focus not just on the learner, the environment or indeed each in isolation, but rather on the changing relationships between these over a given period of time and across different settings’ (McLinden et al. 2016, p. 17). A key challenge for the caregiver in a given early childhood development setting within the child’s *microsystem* (e.g. CBCC in the context of Malawi) is to develop and promote those accommodations that are designed to be both progressive and mutual, through seeking to reduce potential barriers to inclusion within the centre whilst developing and promoting the child’s life skills to encourage them to participate in the activities of the centre and be able to generalise them to home and community settings.

In practical terms, however, it may not be easy to determine how best to recognise or act on children’s preferences for learning and participating in an early childhood development curriculum that may provide limited opportunities for individual needs. A challenge for those engaged in training the caregivers within the child’s *mesosystem* is to find accessible frameworks to support assessment within the educational setting and family settings and prioritise goals and intervention practices that can be applied in low-resource setting. An example of one approach being explored in the Malawi study is the Leuven Scales of Involvement and Well-Being which encourages the adult to consider ways of supporting the child’s levels of ‘engagement’ in a learning or recreational environment (Laevers 2015, p. 2).

Weisner (2002) argues for a nuanced act of imagining a child or infant in a given community and consider the pathways and activities surrounding the child using an ecocultural perspective that takes account of ecological influences. Through such a perspective, shared beliefs and cultural practices can be considered when designing intervention that will help those stakeholders involved in supporting children with disabilities and their families that will better support the family routine and increase the child’s chances of being more accepted by his/her community. As we have argued above, there is a need therefore for greater awareness of the different learning and development needs of children in early childhood development settings and in the communities surrounding the settings, as well as for caregivers to have better knowledge and training to include children with disabilities within different organised daily learning activities.

This raises the question of how a child’s development can best be supported in a context such as rural Malawi and what sorts of inclusive activities can be shown to support their active participation. Given the limited amount of training that is provided to caregivers and interventions that can be offered to children with disabilities, we contend that it is important to consider simple, realistic and achievable goals that will make changes to the lives of children with disabilities. Our initial work in this area highlights that caregivers are provided with few opportunities to assess children’s strengths and needs, keep track of their progress and decide on appropriate strategies to promote learning and development. Therefore, there is a need to provide strategies that are flexible and easy to use considering the low level of resources. Future publications reporting the findings of this study will provide important information for early childhood development research, policy and practice.

## Conclusion

Given the narrow scope and conceptualisation of ‘inclusion’ for young children with disabilities in research studies within LMICs, we have argued in this paper that a bioecological perspective offers the potential for a broader unit of analysis for policy, research and practice in disability studies that is sensitive to different cultural contexts in seeking to optimise individual development across the human lifespan. Situating research studies within the parameters of such a framework therefore enables potential comparison of studies across national boundaries and contexts.

We will draw on the bioecological systems approach framework to help us to interpret and map the data from each stage of the project onto the different systems (micro–macro level) of the adapted model. Researching inclusive early childhood development through such a perspective emphasises the importance of also engaging with different levels of support to ensure appropriate solutions are offered to families who have children with disabilities within a complex ecology. Some of these solutions do not require specific policy changes but do need local communities and services to be committed to seeking workable solutions which have cultural relevance to ensure young children with disabilities can benefit from inclusive and equitable quality early years opportunities so as to positively influence their developmental outcomes.

## References

[CR1] Albrecht GL, Seelman KD, Bury M (2001). Handbook of disability studies.

[CR2] Anderson J, Boyle C, Deppeler J, Zhang H, Wing P, Chan K, Boyle C (2014). The ecology of inclusive education: Reconceptualising Bronfenbrenner. Equality in education: Fairness and inclusion.

[CR3] Artiles AJ, Kozleski EB (2016). Inclusive education’s promises and trajectories: Critical notes about future research on a venerable idea. Education Policy Analysis Archives.

[CR4] Black MM, Walker SP, Fernald LCH, Andersen CT, Di Girolamo AM, Lu C, McCoy DC, Fink G, Shawar YR, Shiffman J, Devercelli AE, Wodon QT (2017). Advancing early childhood development: From science to scale. The Lancet.

[CR5] Bronfenbrenner U (1977). Toward an experimental ecology of human development. American Psychologist.

[CR6] Bronfenbrenner U (2005). Making human beings human: Bioecological perspectives on human development.

[CR7] Coleman M (2013). Empowering family-teacher partnerships: Building connections within diverse communities.

[CR8] Cunningham RD (2004). Delay in referral to early-intervention services. Pediatrics.

[CR9] Ertem IO, Rudolph CD, Rudolph AM, Lister GE, First L, Gershon AA (2011). Monitoring and supporting early childhood development. Rudolph’s pediatrics.

[CR10] Gladstone M, McLinden M, Douglas G, Jolley E, Schmidt E, Chimoyo J, Magombo H, Lynch P (2017). “Maybe I will give some help…. maybe not to help the eyes but different help”: An analysis of care and support of children with visual impairment in community settings in Malawi. Child, Care, Health and Development..

[CR11] Hewett R, Douglas G, McLinden M, Keil S (2017). Developing an inclusive learning environment for students with visual impairment in higher education: Progressive mutual accommodation and learner experiences in the United Kingdom. European Journal of Special Needs Education..

[CR12] International Centre for Evidence in Disability. (2014). *The Malawi key informant child disability project: Summary report*. London, UK: London School of Hygiene and Tropical Medicine. Retrieved from http://disabilitycentre.lshtm.ac.uk/.

[CR13] Laevers F (2015). Making care and education more effective through wellbeing and involvement. An introduction to experiential education.

[CR14] Lynch P, Gladstone M, McLinden M, Douglas G, Jolley E, Schmidt E, Chimoyo J (2018). ‘I have learnt to love the child and give opportunities to play with peers’: A feasibility study of the training programme to support parents of young children with visual impairment in Malawi. Journal of Early Childhood Research.

[CR15] Malawi Government (2008). National syllabus for early childhood development.

[CR16] Malawi Government (2017). Malawi national policy on early childhood development.

[CR17] Malawi National Statistical Office. (2016). *Statistical yearbook.* Zomba, Malawi: Author. Retrieved from http://www.nsomalawi.mw/.

[CR18] McLinden M, Douglas G, Cobb R, Hewett R, Ravenscroft J (2016). ‘Access to learning’ and ‘learning to access’: Analysing the distinctive role of specialist teachers of children and young people with vision impairments in facilitating curriculum access through an ecological systems theory. British Journal of Visual Impairment.

[CR19] McLinden M, McCracken W (2016). Review of the visiting teachers service for children with hearing and visual impairment in supporting inclusive educational practice in Ireland: Examining stakeholder feedback through an ecological systems theory. European Journal of Special Needs Education..

[CR20] McLinden M, Ravenscroft J, Douglas G, Hewett R, Cobb R (2018). The significance of specialist teachers of learners with visual impairments as agents of change: Examining personnel preparation in the United Kingdom through a bioecological systems theory. Journal of Visual Impairment and Blindness.

[CR21] Munthali AC, Mvula PM, Silo L (2008). An inventory of community child care centres in Malawi.

[CR22] Munthali AC, Mvula PM, Silo S (2014). Early childhood development: The role of community based child care centres in Malawi. SpringerPlus.

[CR23] Neuman M, McConnell C, Kholowa F (2014). From early childhood development policy to sustainability: The fragility of community-based childcare services in Malawi. International Journal of Early Childhood.

[CR24] Rogoff B (2003). The cultural nature of human development.

[CR25] Skinner D, Weisner T (2007). Sociocultural studies of families of children with intellectual disabilities. Mental Retardation and Developmental Disabilities Research Reviews.

[CR26] Tudge JR, Mokrova I, Hatfield BE, Karnik B (2009). Uses and misuses of Bronfenbrenner’s bio-ecological theory of human development. Journal of Family Theory & Review.

[CR27] United Nations. (1989). *Convention on the Rights of the Child.* New York: United Nations. Retrieved from http://www.ohchr.org/EN/ProfessionalInterest/Pages/CRC.aspx.

[CR28] United Nations. (2006). *Convention on the Rights of Persons with Disabilities*. New York: UN. Retrieved from www.un.org/disabilities.

[CR29] United Nations. (2015). *Transforming our world: The 2030 agenda for sustainable development.* New York: Division for Sustainable Development Goals, United Nations Secretariat. Retrieved from https://sustainabledevelopment.un.org/sdgs.

[CR30] United Nations Children’s Fund (2012). Integrated social protection systems: enhancing equity for children.

[CR31] Weisner T (2002). Ecological understanding of children’s developmental pathways. Human Development.

[CR32] Werning, R., Artiles, A. J.; Engelbrecht, P., Hummel, M., Caballeros, M. Rothe, A. (2016). *Keeping the promise? Contextualizing inclusive education in developing countries.* Bad Heilbrunn: Klinkhardt. Retrieved from http://www.pedocs.de/frontdoor.php?source_opus=12353&la=de.

[CR33] Woodhead, M., Feathersone, I., Bolton, L., & Robertson, P. (2014). *Early childhood development: Delivering inter*-*sectoral policies, programmes and services in low‐resource settings. Topic guide*. Oxford, UK: Health & Education Advice & Resource Team (HEART). Retrieved from http://oro.open.ac.uk/.

[CR34] World Bank (2015). Protecting early childhood development in Malawi: Baseline report.

[CR35] World Bank Country. (2018). *World Bank list of economies.* Washington DC: World Bank. Retrieved from http://datahelpdesk.worldbank.org/knowledgebase/articles/906519-world-bank-country-and-lending-groups.

[CR36] World Health Organisation (WHO). (2012). *Early childhood development and disability: A discussion paper*. Geneva, Switzerland: WHO. Retrieved from http://apps.who.int/iris/bitstream/10665/75355/1/9789241504065_eng.pdf.

[CR37] Yousafzai A, Lynch P, Gladstone M (2014). Moving beyond prevalence studies: Screening and interventions for children with disabilities in low and middle income countries. Archives of Disease in Childhood.

